# The activation of OR51E1 causes growth suppression of human prostate cancer cells

**DOI:** 10.18632/oncotarget.10197

**Published:** 2016-06-21

**Authors:** Désirée Maßberg, Nikolina Jovancevic, Anne Offermann, Annika Simon, Aria Baniahmad, Sven Perner, Thanakorn Pungsrinont, Katarina Luko, Stathis Philippou, Burkhard Ubrig, Markus Heiland, Lea Weber, Janine Altmüller, Christian Becker, Günter Gisselmann, Lian Gelis, Hanns Hatt

**Affiliations:** ^1^ Department of Cell Physiology, Ruhr-University Bochum, Bochum, Germany; ^2^ Pathology of the University Hospital of Luebeck and the Leibniz Research Center Borstel, Luebeck and Borstel, Germany; ^3^ Institute of Human Genetics, Jena University Hospital, Jena, Germany; ^4^ Institute for Pathology und Cytology, Augusta-Kranken-Anstalt gGmbH Bochum, Bochum, Germany; ^5^ Clinic for Urology, Augusta-Kranken-Anstalt gGmbH Bochum, Bochum, Germany; ^6^ Cologne Center for Genomics, University of Köln, Köln, Germany; ^7^ Present address: Global Drug Discovery - Clinical Sciences, Bayer Pharma AG, Wuppertal, Germany

**Keywords:** OR51E1, proliferation, prostate cancer, cellular senescence, androgen receptor

## Abstract

The development of prostate cancer (PCa) is regulated by the androgen-dependent activity of the androgen receptor (AR). Androgen-deprivation therapy (ADT) is therefore the gold standard treatment to suppress malignant progression of PCa. Nevertheless, due to the development of castration resistance, recurrence of disease after initial response to ADT is a major obstacle to successful treatment. As G-protein coupled receptors play a fundamental role in PCa physiology, they might represent promising alternative or combinatorial targets for advanced diseases. Here, we verified gene expression of the olfactory receptors (ORs) OR51E1 [prostate-specific G-protein coupled receptor 2 (PSGR2)] and OR51E2 (PSGR) in human PCa tissue by RNA-Seq analysis and RT-PCR and elucidated the subcellular localization of both receptor proteins in human prostate tissue. The OR51E1 agonist nonanoic acid (NA) leads to the phosphorylation of various protein kinases and growth suppression of the PCa cell line LNCaP. Furthermore, treatment with NA causes reduction of androgen-mediated AR target gene expression. Interestingly, NA induces cellular senescence, which coincides with reduced E2F1 mRNA levels. In contrast, treatment with the structurally related compound 1-nonanol or the OR2AG1 agonist amyl butyrate, neither of which activates OR51E1, did not lead to reduced cell growth or an induction of cellular senescence. However, decanoic acid, another OR51E1 agonist, also induces cellular senescence. Thus, our results suggest the involvement of OR51E1 in growth processes of PCa cells and its impact on AR-mediated signaling. These findings provide novel evidences to support the functional importance of ORs in PCa pathogenesis.

## INTRODUCTION

Prostate cancer (PCa) is the most common non-cutaneous malignancy and the second leading cause of cancer-related deaths in men of Western countries [1]. Until today, the rate of PCa diagnoses has dramatically increased due to the higher life expectancy of today's population as well as prostate specific antigen [PSA; kallikrein-related peptidase 3 (KLK3)] screening resulting in early detection of PCa. Current therapeutic strategies for advanced stages include primarily androgen-deprivation therapy (ADT). However, after initial regress of the tumor in response to ADT, the disease relapses through the development of castration-resistance [2] by adaptive responses of the AR [3]. Therefore, the demand for effective therapeutic strategies in advanced tumor stages and the discovery of marker genes associated with aggressive PCa continue to increase.

The development of both benign prostatic tissue and PCa is androgen-dependent, although androgens may have diverse effects on cell growth. Depending on the concentration, androgens can promote as well as suppress cell proliferation through the regulation of the androgen receptor (AR) [4–9]. For example, cellular senescence is a natural mechanism to suppress malignant cell growth through the regulation of various tumor suppressor proteins [9–13]. In addition, exogenous and endogenous stimuli cause irreversible cell cycle arrest leading to morphological changes and altered gene expression [14, 15]. This tumor growth suppression is one of the most important downstream mechanisms regulated by the androgen-dependent activity of the AR [9, 16].

However, the progression to castration-resistant prostate cancer (CRPC) leads to more aggressive diseases resulting in metastasis [2, 17]. Although in this case PCa cell physiology is no longer dependent upon exogenous androgens, gene expression is mostly still regulated by the AR. Alternative modulation of the AR by various signaling molecules was previously suggested to be initiated by growth factors, cytokines and GPCRs and thus might influence downstream AR-target gene expression [18–22].

Interestingly, the prostate specific G-protein coupled receptor (PSGR; OR51E2) and its paralog PSGR2 (OR51E1) are overexpressed in PCa [23–27]. Although OR51E1 and OR51E2 are ubiquitously expressed in various types of normal and cancerous tissues in humans [28–31], they are significantly more abundant in the healthy human prostate [28]. Both GPCRs belong to the largest subgroup in the mammalian genome, the olfactory receptor (OR) multigene family. ORs are located predominantly in the cilia of olfactory sensory neurons and detect volatile substances in the environment. However, ORs are not restricted to the olfactory epithelium but are also ectopically expressed in various non-olfactory tissues [31–40]. In the last decade, ectopically expressed ORs have attracted attention since their physiological function has been determined [41–44]. Due to their expression in human cancer tissues, functional studies are the subject of current research aimed at elucidating a possible role for ORs in cancer development and progression [36, 45, 46]. In this context, OR51E2 was the first identified hormone-sensitive membrane receptor involved in the growth mechanisms of PCa cells [45]. The β-ionone-dependent activation of OR51E2 resulted in a reduced proliferation of PCa cells via the Src-Pyk2- p38MAPK signaling pathway [45, 47, 48]. Controversy studies demonstrated prostate cancer growth promoting function of PSGR *in vivo* [49, 50]. Concomitantly, β-ionone stimulation even promotes LNCaP cell invasiveness *in vitro* and metastases spreading *in vivo* [49].

Additional ORs were shown to be involved in the cytokinesis and proliferation of carcinoma cells [36, 46], indicating that they may be possible targets for cancer therapy. Nevertheless, although the OR51E1 receptor has been deorphanized [51], its role in prostate cancer physiology remains unexplored. Because cross-talk between the AR and GPCRs has already been demonstrated [19, 22], we aimed to explore whether the activation of OR51E1 might affect AR downstream signaling and PCa physiology.

Here, we revealed that the treatment with the OR51E1 agonist nonanoic acid (NA) results in the phosphorylation of various protein kinases involved in cellular growth of LNCaP cells. NA reduces androgen-dependent AR-target gene expression and promotes cellular senescence via the Src-p21-E2F1-p38α signaling pathway leading to an inhibition of cell growth. Thus, these findings could significantly contribute to the understanding of OR function in PCa cells, indicate novel signaling towards AR-dependent signaling and provide novel insights of the physiological relevance of OR51E1 in PCa pathogenesis.

## RESULTS

### OR expression profile in human prostate tissue as determined by RNA-Seq

To investigate the gene expression profile of human prostate tissue, RNA-Seq data of benign prostatic and PCa tissues of the human were analyzed generated by the Next Generation Sequencing (NGS)-technique. For this purpose, a publicly available data set obtained from the NCBI GEO database consisting of matched benign prostatic and PCa tissue from ten different patients (P1-P10) was calculated. Additionally, three self-generated data sets of PCa tissues (P11-P13) were analyzed. As represented with a colored scale, FPKM values of 0.1-1 indicate a weak expression level, 1-50 corresponds to a moderate expression level and 50- >1000 illustrates a strong expression level. To ensure a homogenous gene expression and a comparability of all investigated tissues, the distribution of a subset of housekeeping genes [63] and prostate luminal epithelial markers [64] were investigated. All benign prostatic and PCa tissues showed nearly uniform expression levels of the housekeeping genes glyceraldehyde-3-phosphate dehydrogenase (GAPDH), beta-actin (AKTB), chromosome 1 open reading frame 43 (C1orf43), charged multivesicular body protein 2A (CHMP2A) and proteasome subunit beta (PSMB) type 2 and 4, as well as the prostate luminal epithelial marker proteins cytokeratin (KRT) 8 and 18 (Supplementary Figure S1).

**Figure 1:**
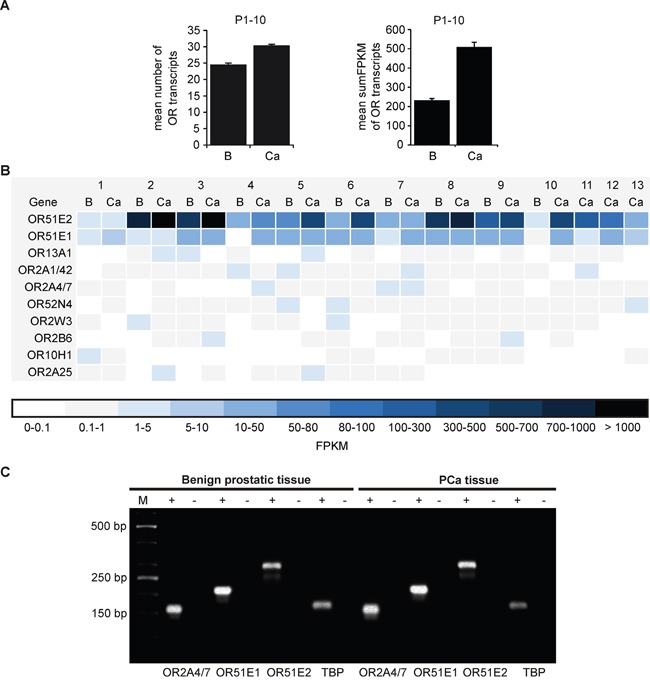
Expression profile of ORs in benign prostatic and PCa tissue as determined by RNA-Seq **A.** (Left) Shown is the average number of expressed ORs with an FPKM >0.1 of all annotated OR genes (n = 387) in human benign prostatic (B) and PCa (Ca) tissues (P1-P10). (Right) Summed FPKM values of all expressed OR genes in human benign prostatic (B) and PCa (Ca) tissues (P1-P10). The FPKM values of the benign prostatic and PCa samples (P1-P10) were averaged. Error bars represent the standard deviation. **B.** The heat map illustrates the ten most highly expressed OR transcripts in human benign prostatic (B) and PCa (Ca) tissues (P1-P13). The RNA-Seq data sets were obtained from ten different patients (P1-P10). In addition three self-generated data sets of prostate PCa tissue were analyzed (P11-P13). Genes in the heat maps are sorted by the sum of all investigated tissues. Black and dark blue represent high transcript expression, and light blue and white indicate low to no detectable transcript expression. **C.** RT-PCR for the verification of OR2A4/7, OR51E1 and OR51E2 in benign prostatic and PCa tissue. The housekeeping gene TATA box binding protein (TBP) was used for quality control of the cDNA. The RNA samples converted into cDNA are shown as (+), and the negative controls containing RNA are shown as (−). M: marker; bp: base pairs.

Using these methods, we investigated the expression profile of all intact OR genes and the average number of expressed ORs with an FPKM >0.1 in benign prostatic and PCa tissue (P1-P10) was calculated. The analysis demonstrated a mean expression of approximately 25 ORs in benign prostatic tissue and approximately 30 ORs in PCa tissue of all 387 intact OR genes with an FPKM >0.1 (Figure 1A, left). Next, the mean sum of all OR FPKM values was calculated. This analysis showed that the mean sum FPKM value in prostate PCa tissue (509.7) is doubled compared to benign prostatic tissue (232.9; Figure 1A, right). Thus, this analysis implies both an increased number of expressed ORs and an increased cumulative expression in PCa.

To compare the OR expression patterns between individual patients, the ten most highly expressed ORs were analyzed (Figure 1B). This analysis confirmed that OR51E2 and OR51E1 are the most highly expressed receptors in nearly all of the investigated prostate tissues. As shown here, OR51E2 exhibits the highest expression with an FPKM value of up to 773 in benign prostatic tissue (P2) and an FPKM value of up to 1133 in PCa tissue (P3). Thus, the expression level of OR51E2 in some patients is comparable to that of the highly expressed housekeeping genes (the mean FPKM value of GAPDH across all tissues examined: 705.2). For the second most highly expressed OR, OR51E1, we observed the highest FPKM value of 34.2 in benign prostatic and 39.7 in PCa tissue (P5). The strongest expression of the third most highly expressed OR, OR13A1, was observed in P5 PCa tissue (FPKM: 3.6). Due to the sequence homology of the ubiquitously expressed OR2A1 and OR2A42 (as well as OR2A4 and OR2A7), these transcripts were considered together as OR2A1/42 (and OR2A4/7) according to Flegel et al. (2013) and registered as the fourth and fifth most highly expressed ORs in prostate tissue. Transcripts are expressed in all of the tissues examined with the exception of the benign prostatic P2-P4 samples. Additional candidates, namely OR52N4, OR2W3, OR2B6, OR10H1 and OR2A25 are expressed in almost all tissues examined. Representatively, the transcript expression of OR51E2, OR51E1 and OR2A4/7 detected by RNA-Seq were verified in benign prostatic and PCa tissue via RT-PCR (Figure 1C).

### Differential expression analysis of OR51E1 and OR51E2 in prostate tissue

**Figure 2:**
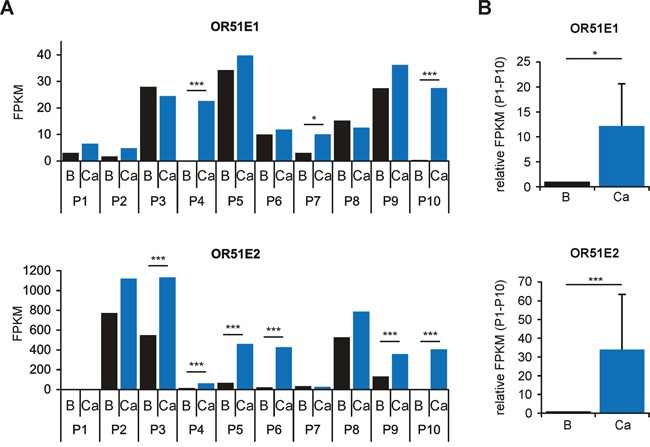
Differential expression analysis of OR51E1 and OR51E2 in human prostate tissue as determined by RNA-Seq **A.** Differential expression analysis of the receptors OR51E1 (top) and OR51E2 (bottom) in benign prostatic (B, black bars) and PCa tissues (Ca, blue bars) from ten different patients (P1-P10). Statistical significances of matched tissue pairs were analyzed using Cuffdiff. **B.** The bar diagram demonstrates significantly increased expression of the receptors OR51E1 and OR51E2 in PCa tissue (P1-P10, Mann-Whitney Rank Sum Test). Each FPKM value for OR51E1 (top) and OR51E2 (below) of benign prostatic (B, black bars) samples (P1-P10) was normalized to the FPKM value of the corresponding PCa samples (Ca, blue bars). Error bars represent the mean ± SEM. * p < 0.05, *** p < 0.001.

To confirm the overexpression of OR51E1 and OR51E2 in PCa as previously described [23–27], we reanalyzed the RNA-Seq data concerning differences in OR gene expression between benign prostatic and PCa tissue. Both receptors showed higher FPKM values in PCa tissues compared with the matched benign prostatic tissues, with the exceptions of OR51E1 in P3 and P8 and OR51E2 in P7 (Figure 2A). Furthermore, we independently analyzed each tissue pair using the statistical Cufflinks application Cuffdiff. This kind of statistics with multiple comparisons revealed that OR51E1 is only highly significantly overexpressed in the PCa tissue of three patients (P4, P7 and P10; Figure 2A and Supplementary Figure S3). For OR51E2, six patients (P3-P6, P9 and P10) exhibit a significant overexpression in PCa tissue (Figure 2A and Supplementary Figure S3). All other intact OR genes did not exhibit significant differences in expression between benign prostatic and PCa tissue.

To illustrate the differences in expression of OR51E1 and OR51E2 between benign prostatic and PCa tissue of the entire group, each FPKM value of PCa tissue was normalized to that of benign prostatic tissue. As demonstrated in Figure 2B, both receptors showed a significantly increased expression level in PCa tissue. Aside from ORs, we also identified genes associated with olfactory signal transduction in the prostate (Supplementary Figure S2) as previously shown for other human tissues [28, 37, 44, 46]. Across all samples examined, adenylyl cyclase 3 (ADCY3) and the olfactory G-protein subunit Gα_olf_ (GNAL) exhibit the highest expression levels, indicating a possible role for cAMP-mediated pathways in the prostate similar to that in olfactory sensory neurons. A lack of expression was observed for the cyclic nucleotide gated ion channel (CNG) subunits A2 and B3 and the receptor transporter protein 2 (RTP2). OR51E2, in contrast, triggers a pathway involving a Src-kinase and the transient receptor potential channel V6 (TRPV6) in LNCaP cells [47]. We identified the gene expression of all members of the Src-kinase family, namely SRC, YES1, FYN, FGR, LCK, HCK, BLK, LYN and FRK, as well as of TRPV6 in human prostate tissue by RNA-Seq (Supplementary Figure S2).

### Protein expression and localization of OR51E1 and OR51E2 in benign prostatic and PCa tissue specimens

**Figure 3:**
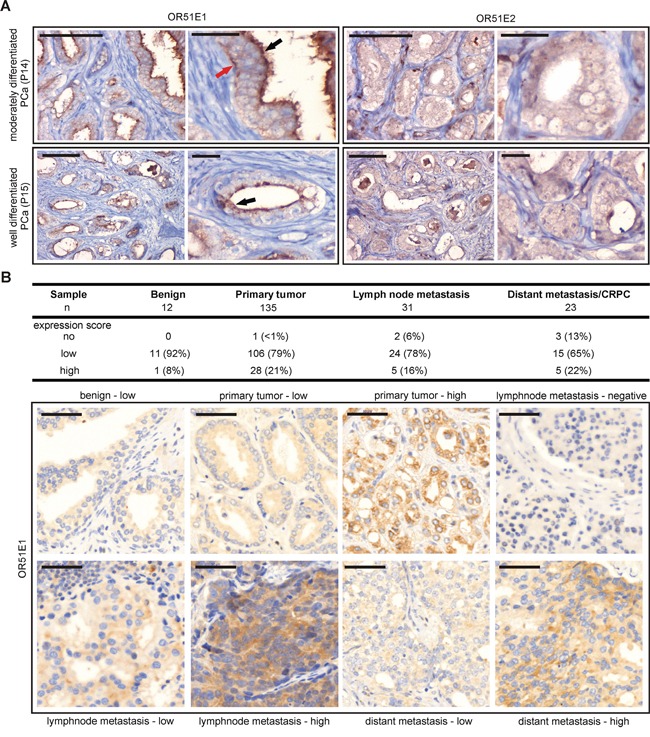
Protein expression of OR51E1 and OR51E2 in human prostate tissue **A.** IHC of prostate tissues using specific human α-OR51E1- and α-OR51E2 antibodies. Shown is the expression of both receptor proteins in prostate epithelial cells of moderately differentiated and well differentiated PCa. The expression of OR51E1 is primarily localized in the apical cytoplasm (black arrow) but is also observed in basal epithelial structures (red arrow) or diffusely in cytoplasm, similar to OR51E2. Protein expression is visualized using DAB chromogenic staining. Tissue architecture is illustrated by co-staining with HE. Scale bar: 100 μm, enlarged: 25 μm. **B.** IHC of a large PCa progression cohort including benign tissue, primary tumors, lymph node metastases as well as distant metastases/CRPC tissues using α-OR51E1 antibody. Upper table shows the expression score of OR51E1 protein in the different prostate tissues indicated as no, low or high expression level. Protein expression is visualized using DAB chromogenic staining and tissue architecture was stained by HE and bluing reagent. Scale bar: 50 μm.

OR51E1 and OR51E2 were previously described to be expressed in the secretory epithelial cells of the prostate [23, 27]. To date, the detection of both ORs was exclusively conducted at the transcript level due to the lack of availability of specific OR-antibodies. To investigate the protein expression of both receptors in prostate tissue, we performed IHC of prostate tissue sections using newly designed α-OR51E1- and α-OR51E2- antibodies. Here, we demonstrated the subcellular OR51E1 and OR51E2 protein expression in prostate epithelial cells of both benign prostatic and PCa tissue with varying differentiation degrees (Figure 3A and 3B). The most intense OR51E1 protein expression is observed in apical luminal cell structures, indicating a membrane localization pattern. However, both receptors also showed expression in basal gland structures and diffusely throughout the cytoplasm of epithelial cells. Due to the heterogeneity of PCa, the protein expression intensities of both receptors vary within the tumor samples. Using the basal cell-specific marker cytokeratin 34βE12, whose presence excludes usual type prostate adenocarcinoma, we were able to distinguish between benign prostatic and PCa tissue (Supplementary Figure S4). To confirm our results, we performed IHC to detect OR51E1 protein on a large PCa progression cohort including benign prostatic tissue, primary tumors, lymph node metastases as well as distant metastases/CRPC. Consistently, OR51E1 expression was heterogeneously within benign prostatic as well as tumor samples and detectable in the cytoplasm and membrane (Figure 3B). Beside primary tumors, OR51E1 was also found to be expressed in 94% lymph node metastases samples and in 87% of distant metastases samples. The specificity of the OR51E1 antibody was demonstrated by IF staining in transfected Hana3A cells expressing a rhodopsin-tagged (rho) OR51E1 construct. These experiments revealed co-staining of both rho and OR51E1 in Hana3A cells (Supplementary Figure S5). Specificity of OR51E2 antibody was equally tested (personal communication, Gelis et al.).

### OR expression analysis in the PCa cell line LNCaP

**Figure 4:**
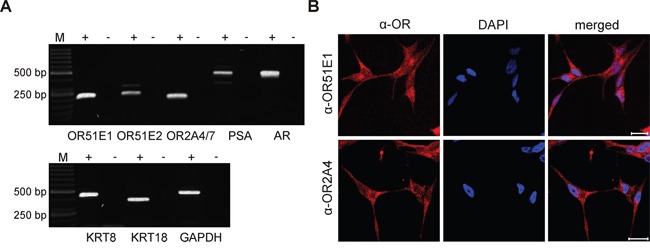
Expression of ORs in the PCa cell line LNCaP **A.** RT-PCR detection of OR51E1, OR51E2 and OR2A4/7 in LNCaP cells. In addition, the prostate specific markers PSA, AR, KRT8 and KRT18 were examined. The housekeeping gene GAPDH was used for quality control of cDNA. The cDNA samples are represented as (+), and the negative controls containing RNA are represented as (−). M: marker; bp: base pairs. **B.** IF detected protein expression of the receptors OR51E1 (top, red) and OR2A4/7 (bottom, red) in LNCaP cells visualized by confocal immunofluorescence microscopy. Nuclei were stained with DAPI (blue). Scale: 20 μm.

Next, we were interested in uncovering the physiological role of OR51E1 in PCa cells. The LNCaP cell line was previously successfully used as a model system to characterize the function of OR51E2, the paralog of OR51E1 [45]. Here, we were able to validate the transcript expression of both OR51E1 and OR51E2 and to identify the transcript expression of OR2A4/7 by RT-PCR in LNCaP cells (Figure 4A). Additionally, RT-PCR analysis showed a positive transcript expression of the prostate luminal epithelial markers PSA, AR, KRT8, and KRT18 (Figure 4A). To confirm the protein expression of OR51E1, OR51E2 and OR2A4/7, IF staining with LNCaP cells was performed. Thereby, the subcellular protein expression of all receptors was observed (Figure 4B and Supplementary Figure S6). As the protein expression of OR51E2 was already documented in LNCaP cells [45], the detection of this receptor served as a positive control. The treatment of LNCaP cells with only the secondary antibody demonstrated specific protein staining (control; Supplementary Figure S6).

### OR51E1 agonist NA causes phosphorylation of intracellular protein kinases

Several studies demonstrated that the activation of ectopically expressed ORs can induce a downstream intracellular calcium influx similar to in olfactory sensory neurons [41, 44, 45, 46]. In the present study, we therefore first investigated whether the activation of OR51E1 leads to an increase in intracellular calcium in LNCaP cells. For this purpose, we examined the effects of the OR51E1-activating ligand NA and its structurally related carboxylic acid agonists OA and DA (personal communication, Jovancevic et al.) on the intracellular calcium concentration using calcium imaging. However, even after 10 minutes of application of the OR51E1 agonists, no change in the intracellular calcium concentration was observed (Supplementary Figure S7). In contrast, the OR51E2 agonist β-ionone [45] induces reproducible calcium responses, which served as a positive control for the viability of LNCaP cells and their ability to be activated. Thus, the OR51E1-initiated signal transduction mechanisms in LNCaP cells seem to proceed without the participation of calcium as a second messenger. Additionally, the canonical cAMP-mediated pathway of ORs could also be excluded, as the stimulation with NA did not increase the intracellular cAMP levels (data not shown).

**Figure 5:**
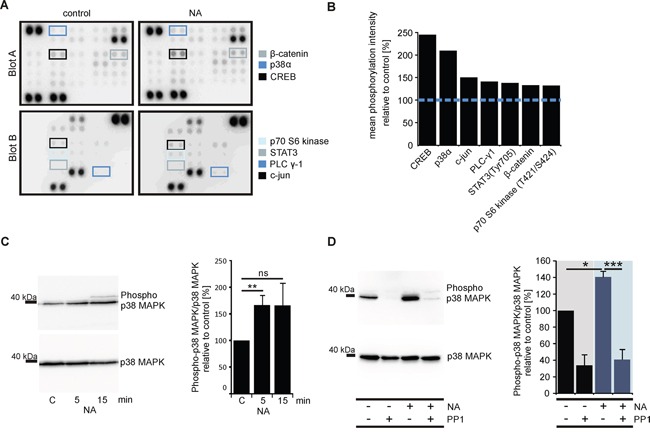
OR51E1 agonist NA induces phosphorylation of protein kinases in LNCaP cells **A.** Nitrocellulose membranes A and B of the Human Phospho-Kinase Array plotted with proteins from the LNCaP cells stimulated with NA (300 μM, right) or control (left). The specific antibodies against phosphorylated protein kinases are spotted in duplicate. The colored boxes marked areas where differences in the protein signal intensities between NA-treated and control-treated cells were detected. **B.** Quantification of the pixel intensities of the phosphorylated protein kinases. The pixel intensities of duplicates were averaged, and the NA-stimulated LNCaP cells were presented relative to the control (blue dashed line, n=1). **C.** Western blot analysis to investigate the phosphorylation levels of p38 MAPK in LNCaP cells after 5 min or 15 min stimulations with NA (300 μM) or control. Determination of the total amounts of p38 MAPK served as controls (left). Quantification of the mean pixel intensities of phosphorylated p38 MAPK relative to p38 MAPK (right). Ratios were normalized against control-treated cells (C) **D.** Western blot analysis to investigate the phosphorylation levels of p38 MAPK in LNCaP cells after 5 min stimulation with NA (300 μM), control, NA along with the Src kinase inhibitor PP1 analog (PP1, 10 μM) and PP1alone (left). The cells were pre-incubated for 30 min with the PP1 analog (10 μM). The total levels of p38 MAPK served as controls. Quantification of the mean pixel intensities of phosphorylated p38 MAPK relative to p38 MAPK (right). Ratios were normalized against control-treated cells. Unless stated otherwise, each Western Blot was repeated in at least three independent experiments. Error bars represent the mean ± SEM. * p < 0.05, ** p < 0.01, *** p < 0.001.

Protein kinases are essential participants in signaling pathways initiated by GPCRs [65]. To further examine potential downstream effector proteins of the OR51E1-initiated signaling pathway, we therefore studied the phosphorylation of 43 different protein kinases using the Proteome Profiler Human Phospho-Kinase Array. LNCaP cells were incubated with NA (300 μM) or control, and the phosphorylation levels of various kinases were determined. Stimulation with NA increased the phosphorylation of 7 kinases by at least 30%: the cAMP response element-binding protein (CREB; 243%), the mitogen-activated protein kinase p38 (p38α, MAPK14; 208%), the proto-oncogene c-jun (149%), the phospholipase C subunit γ1 (140%), the signal transducer activator of transcription 3 (STAT3; 137%), β-catenin (132%) and the p70 S6 kinase (131%) (Figure 5A and 5B). The Human Phospho-Kinase Array indicated that NA drastically increased the phosphorylation of p38α. To confirm these results, Western blot experiments were performed using a specific α-Phospho-p38MAPK antibody. The results showed a significant and reproducible increase in phosphorylation level compared with the control (Figure 5C) but did not show phosphorylation of p42/p44 MAPK or JNK/SAPK (Supplementary Figure S8).

The β-ionone-induced activation of OR51E2 in LNCaP cells triggers a signaling pathway that is mediated by a Src kinase and subsequently initiates MAPK phosphorylation [45, 47]. Therefore, we examined with Western blot experiments whether the NA-induced p38 MAPK activation is mediated by a member of the Src kinase family in LNCaP cells using the selective Src kinase family-inhibitor PP1 analog (4-amino-1-tert-butyl-3- (1′-naphthyl)pyrazolo[3,4-d]pyramidine). The LNCaP cells were stimulated for 5 min in the presence or absence of the PP1 analog along with NA or the control. Subsequently, the phosphorylation level of p38 MAPK was determined as described above. The experiments showed that the phosphorylation was significantly abolished in the presence of the PP1 analog (Figure 5D). Although the PP1 analog reduces the basal activity of p38 MAPK, the NA-dependent increase in p38 MAPK activity was also inhibited.

### NA inhibits proliferation of LNCaP cells and promotes senescence via transcriptional regulation of cell cycle proteins

**Figure 6:**
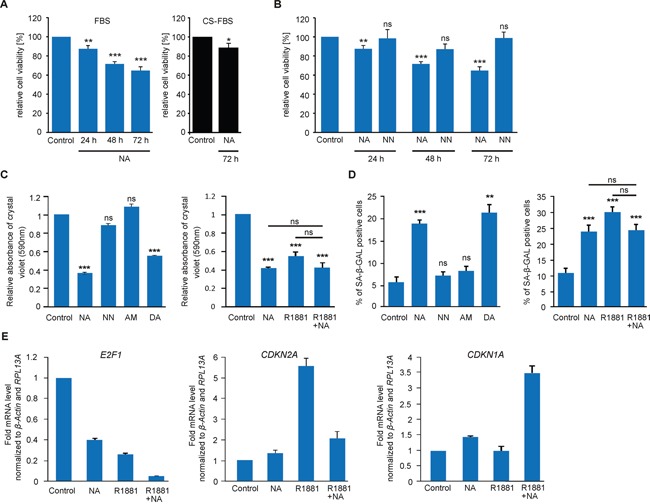
NA reduces the proliferation of LNCaP cells and promotes cellular senescence via the transcriptional regulation of tumor suppressor proteins **A.** MTT proliferation assay of LNCaP cells treated with NA (300 μM) or control for 24 h, 48 h and 72 h in FBS-containing growth medium (10%, blue; n=8) or for 72 h in charcoal-stripped FBS (CS-FBS)-containing medium (10%, black; n=8). All values were normalized to the control, respectively. **B.** MTT proliferation assay of LNCaP cells treated with (left) 300 μM NA, NN, or control for 24 h, 48 h and 72 h to assess the specificity of NA. **C.** Relative density of LNCaP cells measured by crystal violet absorbance after 72 h of treatment with (left) control, 300 μM NA, NN, AM, or DA and (right) control, 300 μM NA, 1 nM R1881, or with the combination of R1881 and NA in growth medium containing 5% FBS (n=6). **D.** Determination of cellular senescence in LNCaP cells after 72 h of treatment with (left) control, 300 μM NA, NN, AM, or DA and (right) control, 300 μM NA, 1 nM R1881, or the combination of R1881 and NA in growth medium containing 5% FBS. LNCaP cells were detected for SA β-Gal activity. Unless stated otherwise, each experimental set contained triplicates and was repeated in at least three independent experiments. Error bars represent the mean ± SEM. * p < 0.05, ** p < 0.01, *** p < 0.001, ns=not significant. **E.** qRT-PCR to estimate relative mRNA expression levels of E2F1 (left), CDKN2A (p16, middle), and CDKN1A (p21, right). LNCaP cells were treated with NA (300 μM), R1881 (1 nM), the combination of both or control. The Ct-values of β-actin and RPL13A were used for normalization.

We have previously shown that the OR-dependent activity of p38 MAPK can result in altered cellular growth [44, 45, 46]. To examine the physiological long-term effects of NA, we initially investigated whether the viability of LNCaP cells is influenced. Using the MTT assay, we observed a significant time-dependent inhibition of proliferation within 24h of stimulation with NA in androgen-containing culture medium (10% FBS). The antiproliferative effect is androgen-independent, because NA also significantly reduced cell proliferation under androgen-free conditions using charcoal-stripped FBS (Figure 6A). In contrast, treatment with the structurally related odorant NN did not cause inhibition of cell growth and therefore served as specificity control (Figure 6B). We additionally performed crystal violet staining of LNCaP cells after 72 h of ligand treatment. These additional experiments demonstrated that NA and another OR51E1 agonist, DA, reduced LNCaP cell growth density by more than 50%, whereas NN and the OR2AG1 agonist AM [66] had no effect (Figure 6C). As androgens are known to possess concentration-dependent antiproliferative potential at supraphysiological levels [9, 67], the synthetic androgen R1881 served as a positive control in the present study. A synergism between NA and R1881 was not observed.

To examine if the inhibition of proliferation is associated with cellular senescence, we measured the senescence-associated *beta-galactosidase* (SA β-Gal) activity in LNCaP cells. The data demonstrated that NA enhances the activity of SA β-Gal, indicating the induction of a cell cycle arrest (Figure 6D). The NN and AM negative controls did not affect either cell number or cellular senescence. In addition, DA induced cellular senescence to a level similar to NA (Figure 6D). As a positive control, we used the less metabolizable androgen R1881 that also induces cellular senescence [9]. No synergism was observed between R1881 and NA. Taken together, our data suggests that the activation of OR51E1 inhibits the growth of PCa cells through cellular senescence.

Next, we assessed the relative gene expression levels of the key regulatory molecules E2F1, p21 (CDKN1A) and p16 (CDKN2A) involved in cellular senescence [9]. qRT-PCR experiments showed reduced mRNA levels of E2F1 upon administration of NA for 48 h (Figure 6E). This effect is even potentiated in the presence of R1881. Additionally, we observed enhanced mRNA levels of the tumor suppressor p16 in the presence of androgens (Figure 6E). Interestingly, the levels of p16 and p21 respond distinctly to the co-treatment of androgens and NA: while the levels of p16 mRNA are reduced compared with agonist alone, the levels of p21 mRNA are enhanced. This suggests that NA might interfere with the activated AR but might also in some cases synergize with AR signaling. Thus, together with the inhibitory effect on proliferation, our results indicate that NA might modulate AR downstream signaling.

### NA affects AR-mediated signaling

**Figure 7:**
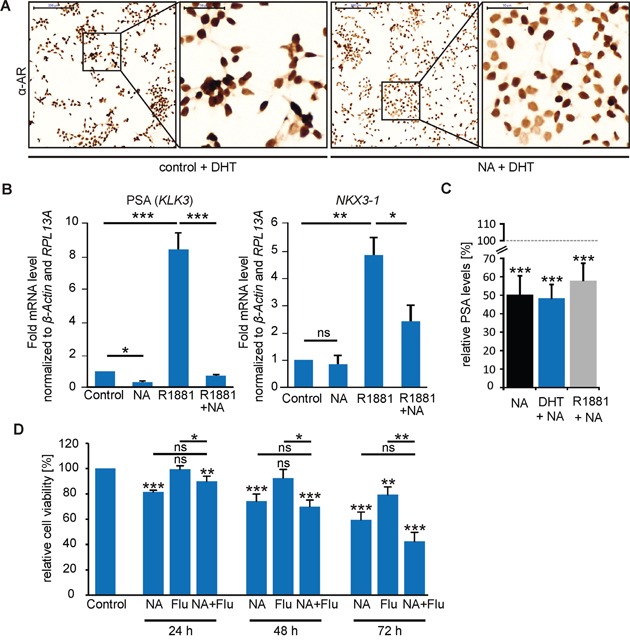
NA affects AR signaling in LNCaP cells **A.** ICC of the AR in LNCaP cells treated with NA (300 μM) or control in the presence of DHT (10 nM). Protein expression is visualized using DAB chromogenic staining. Scale bar: 200 μm (10x); enlarged: 50 μm (40x). **B.** Activation of OR51E1 influences AR-target gene expression. qRT-PCR experiments to estimate relative mRNA expression levels of (left) PSA (KLK3) and (right) the homeobox protein NKX3-1. LNCaP cells were treated with NA (300 μM), R1881 (1 nM), the combination of both or control. The Ct-values of β-actin and RPL13A were used for normalization. **C.** Determination of the PSA levels secreted by LNCaP cells. Cells were treated for 48 h with NA (300 μM) in presence of DHT (10 nM, blue), R1881 (1 nM, gray) or in androgen-free medium (black). PSA levels were presented relative to the same conditions without NA, respectively, as indicated as gray dashed line. Each experimental set contained duplicates and was repeated in three independent experiments. **D.** MTT proliferation assay of LNCaP cells treated with 300 μM NA, flutamide (Flu 10 μM), control or the co-treatment of NA and flutamide (NA + Flu) for 24 h, 48 h and 72 h in 10% FBS-containing medium. Unless stated otherwise, each experimental set contained triplicates and was repeated in at least three independent experiments. Error bars represent the mean ± SEM. * p < 0.05, ** p < 0.01, *** p < 0.001, ns = not significant.

To ascertain a possible influence of NA on AR signaling, we performed ICC to investigate the protein expression and subcellular localization of AR upon NA stimulation in LNCaP cells. We found that NA causes a decrease in the protein abundance in the presence of the androgen DHT after 24 h (Figure 7A). Accordingly, qRT-PCR demonstrated a NA-mediated reduction in mRNA expression levels of the AR-regulated target gene PSA but not of the prostate homeobox protein coding tumor suppressor gene NKX3-1 (Figure 7B). Co-treatment of LNCaP cells with NA and the androgen R1881 resulted in significantly reduced mRNA levels of both genes compared with R1881 treatment alone. These results could indicate an influence of OR51E1-induced signaling on androgen-mediated AR-target gene expression. To strengthen our hypothesis, we measured PSA protein levels in LNCaP medium supernatants. Results of this study showed that NA decreased both the basal PSA level in the absence of androgens, and that due to androgen (DHT or R1881)-enhanced secretion, by nearly 50% (Figure 7C). Additionally, when blocking AR signaling with the known AR antagonist flutamide, the antiproliferative activity of NA was not significantly altered, but slightly enhanced after 72 h of co-treatment (Figure 7D). In summary, we identified that OR51E1 activation reduces proliferation and promotes cellular senescence of LNCaP cells by affecting AR-mediated signaling.

## DISCUSSION

The difficulty of nearly incurable CRPC diseases today necessitates more effective therapies. As GPCRs play a fundamental role in the development of advanced PCa, they might function as alternative targets for pharmacological treatment [22, 68, 69]. In the present study, we were able to elucidate the function of an OR, namely OR51E1 (PSGR2; Dresden-GPCR), previously discussed as a potential biomarker for malignant prostate tissue [27, 70]. Its paralog, OR51E2 (PSGR), was previously identified as an important target that inhibits the proliferation of PCa cells [45].

Herein, the analysis of RNA-Seq data generated by Next Generation Sequencing confirmed the expression of both OR51E1 and OR51E2 and uncovered the expression of additional ORs in human prostate tissue. We identified significantly elevated expression levels of both ORs in the PCa tissue by the mean of all patients. However, strong interindividual variations in receptor expression were identified in benign prostatic tissues and in some PCa specimens no overexpression was observed. Due to the varying OR gene expression levels reflected by FPKM values for OR51E2 ranging from 3.1 to 773 in benign prostatic tissue and 4.6 to 1133.3 in PCa tissue, the mean FPKM for each gene was associated with higher p-values. Similar expression levels of KRT8, KRT18 and housekeeping genes across all patient specimens exclude the possibility of quality differences in the raw data. The interindividual variations in gene expression levels are most likely due to variability in the developmental origins of each tumor, which is responsible for the majority of cancer heterogeneity [71]. Interestingly, the detection of cancer-related genes was associated with strong interindividual variations in benign prostatic tissues as previously shown for OR51E2 [72]. This implies that both receptors could play an important role not only in the normal prostate physiology but also in cancer development and progression. However, whether both receptors are diagnostic tumor markers remains disputable due to their frequently prominent presence also in benign prostatic tissue as well as other human cancer tissues [26–31]. Extensive qPCR studies of a large cohort previously demonstrated significantly elevated expression levels of both receptors [24–27, 70]. As demonstrated here, individual specimens sporadically exhibit contradictory expression pattern which might be due to tumor heterogeneity within the biopsy, tumor subtype and progression or individual expression variations. Therefore, the use as sole tumor markers in prostate cancer diagnosis is unreliable, but they might be applied in combination with further prognostic markers.

Furthermore, we uncovered the expression of additional ORs in the prostate tissue, although no significant differences in gene expression levels were observed between benign prostatic and PCa tissue. Interestingly, the fifth most highly expressed OR, OR2A4 (OR2A4/7), was previously shown to be involved in the cytokinesis of cervical PCa cells [36]. Therefore, a physiological function for this OR in PCa cells is conceivable. Despite the fact that OR2A7 and ARHGEF34P (loc728377) share regions of the same exon [28], we were able to demonstrate the protein expression of this receptor in LNCaP cells. However, the specific agonist is currently unknown, precluding studies to explore its physiological function in the present study. Nevertheless, because OR2A4/7 is also expressed in LNCaP cells, this cell line could serve as a representative model system to characterize the function of this OR in future studies.

Here, we showed the subcellular protein localization of OR51E1 and OR51E2 in benign prostatic and PCa tissues with varying differentiation degrees. While OR51E2 protein is diffusely expressed throughout the cytoplasm of prostate epithelial cells, OR51E1 is primarily localized in the apical cytoplasm but also in basal gland structures, indicating membrane localization. Interestingly, we found OR51E1 protein expression in almost all lymph node and distant metastasis specimens examined, indicating a physiological function of OR51E1 also in advanced PCa.

In the present study, we demonstrated an NA-initiated signaling pathway independent of intracellular calcium elevation. However, in LNCaP cells, NA increased the phosphorylation levels of several protein kinases that are important for cellular growth processes. In this context, we identified an additional ectopically expressed OR that inhibits cancer progression *in vitro* via p38 MAPK signaling. Thus, p38 MAPK seems to be an important regulatory component of OR-mediated effects on cell proliferation outside of nasal tissue, as we previously demonstrated in several *in vitro* systems [44, 45, 46]. Furthermore, various p38 MAPK subtypes are able to interact with the transcription factors c-jun [73], CREB [74, 75, 76], and STAT3 [77] all of which showed increased phosphorylation levels upon NA stimulation. However, the simultaneous induction of a parallel pathway is possible, since Rodriguez et al. (2014) and Sanz et al. (2014) demonstrated a promoted tumor progression via the paralogous receptor PSGR *in vivo*, involving another signaling pathway than p38 MAPK. *In vivo* studies would clarify this central issue in future studies.

NA promotes the growth arrest of androgen-sensitive LNCaP cells involving the induction of cellular senescence. This process seems to be mediated through a crosstalk between protein kinases and various cell cycle regulators, because we observed altered expression of E2F1, p16, and p21. These molecules are hypothesized to be common interaction partners in oncogene-induced senescence mechanism [78]. The p38α-induced senescence represents supporting evidence that this subtype primarily functions in OR-mediated growth inhibition [78]. Furthermore, the induction of cellular senescence is found to be mediated through plasma membrane scaffolds such as GPCRs [79], indicating that OR51E1 could act via mechanisms similar to other clinically relevant GPCRs.

Aside from NA, we demonstrated that the structurally similar DA (capric acid) inhibits the proliferation and promotes the senescence of PCa cells. As primary constituents of coconut and palm oils, bovine milk and other natural products, medium-chain fatty acids are ingested daily with food [80]. Thereby, DA, OA (caprylic acid) and other structurally related medium-chain fatty acids were found to be contained in human blood plasma [81], even in the micromolar range. These concentrations are sufficient to activate OR51E1 (personal communication, Jovancevic et al.). Therefore, DA or more potent fatty acids may represent endogenous activating OR51E1 agonists in human prostate cells. Studies have recently demonstrated that OA and DA possess anticarcinogenic properties in human colorectal, skin and breast cancer cells by regulating cell cycle proteins [82]. Because OR51E1 is ubiquitously expressed throughout the human body [28], it might regulatory function in cellular growth mechanisms in general.

Proliferation experiments under androgen-free conditions demonstrated that the NA-dependent growth suppression of LNCaP is independent of androgens. However, the negative influence on cell growth seems to be enhanced by pre-stimulation with androgens, since the growth suppression was not as pronounced as in comparison to the relative viability of NA-treated cells in serum-containing medium. It cannot be excluded, that simultaneously acting substances such as hormones, growth factors or cytokines synergistically influence growth of LNCaP, particularly substances known to possess the capability to modulate the AR [18–22].

Nevertheless, NA influences androgen-regulated AR target gene expression accompanied by reduced PSA protein levels, and AR signaling is mostly (hyper-) activated in PCa [83]. Furthermore, we observed reduced AR protein expression upon NA in LNCaP. Therefore, an interference with AR signaling might be one possible reason. However, the detailed mechanism by which the NA-signaling could lead to the induction of cellular senescence and inhibited proliferation remains unclear. One hypothesis might be that NA suppresses AR signaling by affecting e.g. AR transcript expression, translation or phosphorylation. A direct interaction of NA signaling components on the AR ligand binding domain seems unlikely because concomitant AR blockade by flutamide did not affect NA-induced inhibition of LNCaP cell proliferation. On the contrary, NA tends to enhance the flutamide inhibitory effect on proliferation, which raises the potential that both substances exert synergistic effects. Further experiments are needed to explore the molecular basis of a possible interaction between OR and AR signaling, e.g. luciferase reporters for AR responsive genes, immunoprecipitation for AR and OR downstream molecules, AR degradation or stability.

The potential suppression of AR signaling may further depend on increased p21 activity that inhibits nuclear AR translocation as previously described [84]. Furthermore, the OR51E1 agonist NA possesses the ability to reduce 5α-reductase activity and thus has anti-androgenic potential [85]. This potential might further enhance the NA-mediated effects observed in our study.

Here, our results provide novel insights into the antiproliferative activity of OR51E1 expressed in PCa cells and support the importance of ORs in PCa pathogenesis. These basic research data might represent the fundament for the identification of possible alternative therapeutic targets for the treatment of advanced PCa.

## MATERIALS AND METHODS

### Ethics statement

Usage of prostate tissue specimens was approved by the Regional Research Ethics Committee (Ethics Committee of the Medical Faculty of Rheinische Friedrich-Wilhelms-University of Bonn). All subjects provided written informed consent to participate. The study was conducted according to the Declaration of Helsinki Principles.

### Solutions and chemicals

All chemicals were purchased from Sigma-Aldrich (Munich, Germany) unless otherwise stated. Octanoic acid (OA), NA and decanoic acid (DA) were obtained from Symrise AG (Holzminden, Germany). The inhibitor 4-Amino-1-tert-butyl-3-(1′-naphthyl)pyrazolo[3,4-d]pyrimidine (PP1 analog) was purchased from Merck Millipore (Darmstadt, Germany). All substances were pre-diluted in DMSO (to a maximal final concentration of 0.1%). Each odorant-DMSO stock solution was dissolved and vortexed in pre-warmed cell culture medium (37°C) or extracellular solution to its final concentration, respectively, without exceeding the maximal water solubility. In all experiments, 0.1% DMSO served as the control.

### Cell culture and human prostate tissue specimens

The LNCaP cell line was purchased from the Leibniz Institute DSMZ - German Collection of Microorganisms and Cell Cultures (Braunschweig, Germany) and cultured in RPMI 1640 medium (Gibco®; Life Technologies, Carlsbad, CA, USA) supplemented with 10% fetal bovine serum (FBS; Gibco®/Sigma-Aldrich), 2 mM L-glutamine and penicillin (100 U/ml) and streptomycin (100 μg/ml; Gibco®) unless otherwise stated.

LNCaP cells [52] used for qRT-PCR, cellular senescence and crystal violet staining were cultured in RPMI 1640 medium supplemented with 5% normal FBS, penicillin (100 U/ml), streptomycin (100 μg/ml), 25 mM HEPES (pH 7.8) and 1% sodium pyruvate. The cells were cultured in a 5% CO_2_ incubator (HERACELL 240i) at 37°C. Depending on the experimental approach, LNCaP cells were treated with the stimulus or control in RPMI 1640 medium containing 0%, 5% or 10% FBS or charcoal-stripped FBS (Thermo Fisher Scientific, Darmstadt, Germany).

Hana3A, a HEK293-derived cell line stably expressing RTP1L, RTP2, REEP1, and Gα_olf_, which supports the heterologous expression of ORs [53] was kindly provided by Prof. H. Matsunami (Duke University Medical Center, Durham, N.C., USA). The cells were cultured in DMEM (Gibco®) supplemented with 10% FBS and penicillin (100 U/ml) and streptomycin (100 μg/ml) and maintained as described above.

Benign and PCa tissue samples were derived from patients undergoing radical prostatectomy, transurethral resection of the prostate or punch biopsies (for clinical data see Supplementary Table S1). Samples were immediately stored at −80°C for subsequent total RNA isolation or immunohistochemistry (IHC) as described below.

The PCa progression cohort examined in IHC comprises 12 benign prostatic tissues, 135 primary PCa, 31 lymph node metastases, and 23 CRPC (90 local recurrent CRPC from the University Hospital of Bonn).

### Total RNA isolation and reverse transcription polymerase chain reaction (RT-PCR)

Total RNA was extracted from LNCaP cells using the RNeasy® Mini Kit (Qiagen, Hilden, Germany) according to the manufacturer's instructions. Benign prostatic and PCa tissue samples were homogenized in lysis buffer prior to RNA extraction. Total RNA concentration and quality (A260/A280 ratio) were analyzed using the NanoDrop ND-1000 Spectrophotometer (Thermo Scientific, Waltham, MA, USA). After DNaseI treatment with the TURBO DNA-free™ Kit (Life Technologies), complementary DNA (cDNA) was synthesized using the iScript™ cDNA Synthesis Kit (Bio-Rad Laboratories, Hercules, CA, USA). RT-PCR was performed under standard conditions using an annealing temperature of 60°C. PCR was performed with cDNA (the equivalent of 50 ng of total RNA) and the specific primer sequences listed in Supplementary Table S2. We used RNA controls (−RT) to exclude contamination with genomic DNA. The resulting products were confirmed using Sanger sequencing.

### Quantitative reverse transcription PCR (qRT-PCR)

The cells were treated for 48 h with control, 1 nM R1881 or 300 μM NA. qRT-PCR and primers used for the detection of E2F transcription factor 1 (E2F1), cyclin-dependent kinase inhibitor 2A (CDKN2A; p16) and cyclin-dependent kinase inhibitor 1A (CDKN1A; p21) were previously described [9, 54]. NK3 homeobox 1 (NKX3-1) and PSA (KLK3) were also analyzed. The obtained data were normalized to the housekeeping genes, β-actin and 60S ribosomal protein L13a (RPL13A).

### Transfection and expression plasmids

For immunofluorescence staining (IF) with heterologously expressed ORs, Hana3A cells were transiently transfected with OR51E1, OR51E2- or OR2A4-pCI constructs using Lipofectamine® 2000 Transfection Reagent (Life Technologies). Rho-tagged pCI expression vectors coding for OR51E1, OR51E2 and OR2A4 were constructed using standard PCR methods as previously described [53].

### RNA-seq analysis

RNA-Seq analyses were performed as previously described [28]. The data from samples P1-P10 used in this study were obtained from the NCBI GEO database (GSE22260). For the sequencing of samples P11-P13, total RNA from prostate tissue was extracted, and mRNA-Seq libraries were prepared with the TruSeq™ RNA Sample Prep Kit v2 protocol for standard mRNA-Seq (Illumina, San Diego, USA). RNA-Seq was performed on the HiSeq 2000 (2×101 bp reads) sequencing platform. RNA-Seq analyses of these samples generated approximately 6.4 million reads. The raw sequence data were aligned to the human genome reference sequence (hg19) using TopHat v2.0.6 [55]. Cufflinks v1.3.0 was used to calculate the FPKM (fragments per kilobase of exon per million fragments mapped) values [56]. Differential expression analyses between benign prostatic and PCa tissue were performed with the Cufflinks application Cuffdiff v.1.3.0 [57]. The Integrative Genomics Viewer v2.3 was used for quality control of the aligned data [58].

### Immunocytochemistry (ICC) and immunohistochemistry (IHC)

The following primary antibodies were used: custom-made affinity purified rabbit IgG polyclonal antibodies against OR51E1 (epitope: LRLFHVATHASEP) and OR51E2 (epitope: ISCDKDLQAVGGK) (Eurogentech, Seraing, Belgium), a rabbit polyclonal anti-OR2A4 antibody (Abcam; Cat. no.: ab97486; dilution: 1:100), Confirm anti-keratin (34ßE12) mouse monoclonal primary antibody (Ventana Medical Systems, Roche, Tucson, AZ, USA), mouse monoclonal anti-AR antibody (AR441, Dako, Glostrup, Denmark; dilution: 1:100), and mouse monoclonal α-Rhodopsin 4D2 antibody (Merck-Millipore, Darmstadt, Germany; dilution: 1:100). IF staining of transfected Hana3A cells and LNCaP cells was performed as previously described [46]. ICC of AR in LNCaP cells was performed according the published protocol for cultured cell lines by Cell Signaling Technology (Cell Signaling Technology, Danvers, MA, USA).

IHC of human prostate tissue paraffin sections (P14 and P15; 4 μm) was performed on a Ventana BenchMark Ultra instrument (Ventana Medical System, Tuscon, AZ) using the UltraView Universal DAB Detection Kit according to the manufacturer's instructions. Primary antibodies were diluted in antibody diluent and incubated as follows: α-OR51E2 (1:50; 32 min), α-OR51E1 (1:30; 32 min) and CK34βE12 (undiluted, 16 min). Additionally, all sections were co-stained with haematoxylin (HE) to illustrate tissue architecture. The staining was visualized using an Olympus BX 43 microscope.

IHC staining of the prostate cancer progression cohort was performed on sections of paraffin embedded tissues using Ventana XT immunostainer (Ventana Medical System). Briefly, slides were incubated with the primary antibody against α-OR51E1 at room temperature following detection as described above. Afterwards, slides were counterstained with HE and bluing reagent, dehydrated and mounted. Cytoplasmic staining level of α-OR51E1 is reflected as an expression score indicated as no, low, and high staining. Cytoplasmic staining intensity of OR51E1 in prostate tissues was qualitatively interpreted by at least two independent pathologists. Tissues with no staining were defined as “negative”, and tissues with positive staining in at least 20% of cells were scored in “low” or “high” staining intensity.

### Human phospho-kinase array

For the detection of the relative phosphorylation levels of intracellular kinases, the Proteome Profiler Human Phospho-Kinase Array Kit (Cat. no. #ARY003, R&D Systems, Wiesbaden, Germany) was used according to the manufacturer's instructions. In general, LNCaP cells were treated for 5 min with NA (300 μM) or control and lysed in Lysis Buffer 6 (R&D Systems). The cell lysates were incubated overnight on the array membrane. The protein levels were quantified using the Java-based ImageJ 1.46 software [59]. Signal differences were evaluated when phosphorylation intensities of NA-stimulated cells showed an increase of at least 30% in comparison with the control. For illustration, the relative pixel intensities of the NA-stimulated samples were normalized to the relative pixel intensities of the control-treated samples.

### Western blot analysis

The following primary antibodies were used: rabbit polyclonal antibodies against p44/42 MAPK (Cat. No. #9102), p38 MAPK (Cat. No. #9212), and SAPK/JNK (Cat. No. #9252), and rabbit polyclonal antibodies against phosphorylated p44/p42 MAPK (Cat. No. #9101), p38 MAPK (Cat. No. #9211) and SAPK/JNK (Cat. No. #9251) (Cell Signaling Technology). LNCaP cells were treated for 5 or 15 min with NA (300 μM), control, NA under administration of the inhibitor PP1 analog or the inhibitor alone. Western blot analyses were performed as previously described [45]. The detection was performed using the ECL™ Select Western Blotting Detection System (Amersham Biosciences, GE Healthcare, Solingen, Germany). The data were quantified using ImageJ 1.46. The relative pixel intensities were calculated by dividing the average pixel intensities of the phosphorylated protein bands by the average pixel intensities of the unphosphorylated protein bands. The relative pixel intensities of the NA-stimulated samples were normalized to the relative pixel intensities of the control-treated samples.

### Calcium imaging

LNCaP cells cultured in 35-mm cell culture dishes were incubated in a standard extracellular solution with 3 μM fura-2-acetoxymethyl ester (Molecular Probes, Eugene, Oregon, USA) for 30 minutes at 37°C in a 5% CO_2_ humidified atmosphere. Fluorometric imaging was performed as previously described [45]. The cells were exposed for 10 minutes to up to 1 mM OA, NA, DA or 500 μM β-ionone using a specialized microcapillary application system. The pre-diluted substances were dissolved in the extracellular solution to the desired concentration.

### PSA ELISA

LNCaP cells were exposed to 300 μM NA or control for 2 days in androgen-free RPMI 1640 medium or in RPMI 1640 medium containing dihydrotestosterone (DHT, 10 nM) or methyltrienolone (R1881; 1 nM). The culture supernatants from the LNCaP cells were collected, centrifuged to remove any residual cells and diluted 1:1 in RPMI medium. The levels of secreted PSA in the cell culture supernatants were determined using the PSA ELISA Kit supplied by Abnova (Cat. no.: KA0208, Taipei, Taiwan) according to the manufacturer's instructions. The PSA levels were measured spectrophotometrically at 450 nm.

### Cell proliferation

LNCaP cells were cultured in RPMI 1640 medium supplemented with 10% FBS (Sigma-Aldrich, St Louis, MO, USA), non-essential amino acids solution (Gibco®), penicillin (100 U/ml), streptomycin (100 μg/ml), 25 mM HEPES (pH 7.8), and 1% sodium pyruvate. The proliferation of LNCaP cells was investigated using MTT (3-(4,5-dimethylthiazol-2-yl)-2,5-diphenyltetrazolium bromide) assay (Roche, Mannheim, Germany) according to manufacturer's instructions. Proliferation was measured 24, 48 and 72 h after cell treatment with 300 μM NA, 1-nonanol (NN) or control. Each experiment was independently repeated three times unless otherwise stated, and within each experiment, each condition was performed in triplicate.

### Crystal violet staining

The crystal violet staining of LNCaP cells was performed as described by Moehren et al. [60] after analyzing the cellular senescence assays. The stained cells were solubilized with Sörenson's solution as described by Jansson et al. (2012) [61]. The solubilized crystal violet was measured for an absorbance at 590 nm with a UV/Vis spectrometer UV3.

### Senescence-associated β-galactosidase (SA β-Gal) staining

The cells were treated for 72 h with control, 1 nM R1881, 300 μM NA, NN, amyl butyrate (AM) or DA. A high concentration of the AR agonist R1881 was used as a positive control for the induction of cellular senescence in LNCaP cells [9]. The senescence-associated β-galactosidase (SA β-Gal) staining was performed as previously described [9, 54, 62].

### Statistics

All of the results were tested for normality (Shapiro-Wilk test) and equal variance. For analyses passing the tests, we used a two-tailed unpaired t-test. We performed the Mann-Whitney Rank Sum Test on data that were not normally distributed.

For the statistical analysis of RNA-Seq data, Cuffdiff was used to compare differences in single matched tissues. For statistical analysis and illustration of the entire group (P1-P10), the FPKM value for an examined gene was normalized to the FPKM value obtained from the benign prostatic tissue of each donor and the group was analyzed by the Mann-Whitney Rank Sum Test. The values represent the mean ± SEM (standard error of the mean) from at least three independent experiments, unless stated otherwise. The statistical significance is indicated in the figures as follows: * p< 0.05, ** p< 0.01, *** p< 0.001.

## SUPPLEMENTARY FIGURES AND TABLES




