# Supplementary material for: The activation of OR51E1 causes growth suppression of human prostate cancer cells
Source: Oncotarget. 2016 Jun 21;7(30):48231–49. doi: 10.18632/oncotarget.10197 (PMC5217014; doi:10.18632/oncotarget.10197)
Supplement: Supplementary file 1 [file oncotarget-07-48231-s001.pdf]

The activation of OR51E1 causes growth suppression of human prostate cancer cells

SUPPLEMENTARY FIGURES AND TABLES

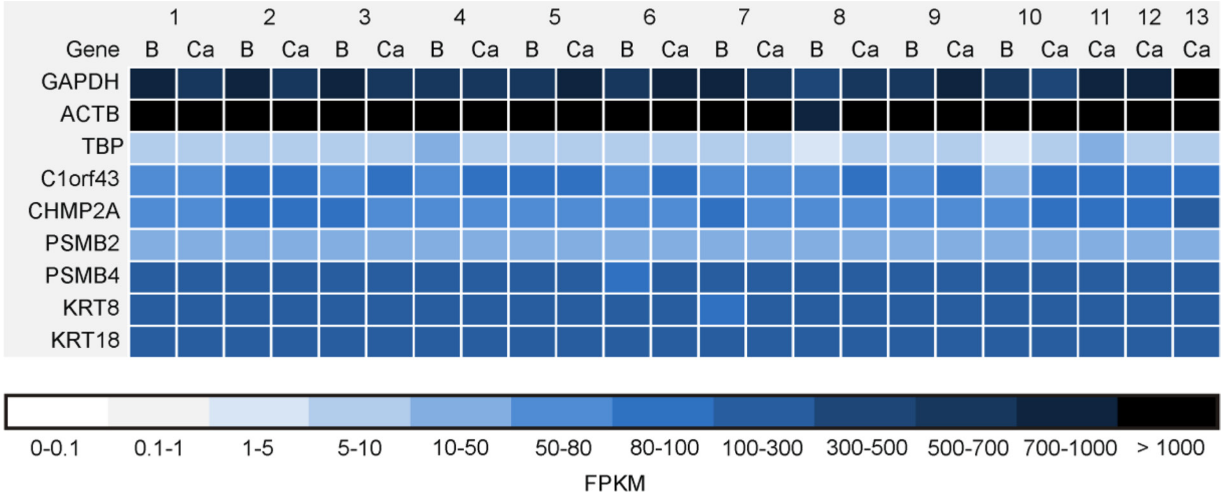

Supplementary Figure S1: Expression profile of housekeeping genes in benign prostatic and PCa tissue as determined by RNA-Seq. The heat map shows the similar expression distribution of the housekeeping genes glyceraldehyde-3-phosphate dehydrogenase (GAPDH), beta-actin (AKTB), chromosome 1 open reading frame 43 (C1orf43), charged multivesicular body protein 2A (CHMP2A), and proteasome subunit beta (PSMB) type 2 and 4 and the prostate luminal epithelial marker proteins cytokeratin (KRT) 8 and 18 in all investigated human benign prostatic (B) and PCa (Ca) tissues (P1-P13). The RNA-Seq data sets were obtained from ten different patients (P1-P10). In addition, three self-generated data sets of PCa tissue were analyzed (P11-P13). Black and dark blue represent high transcript expression, and light blue and white indicate low to no detectable transcript expression.

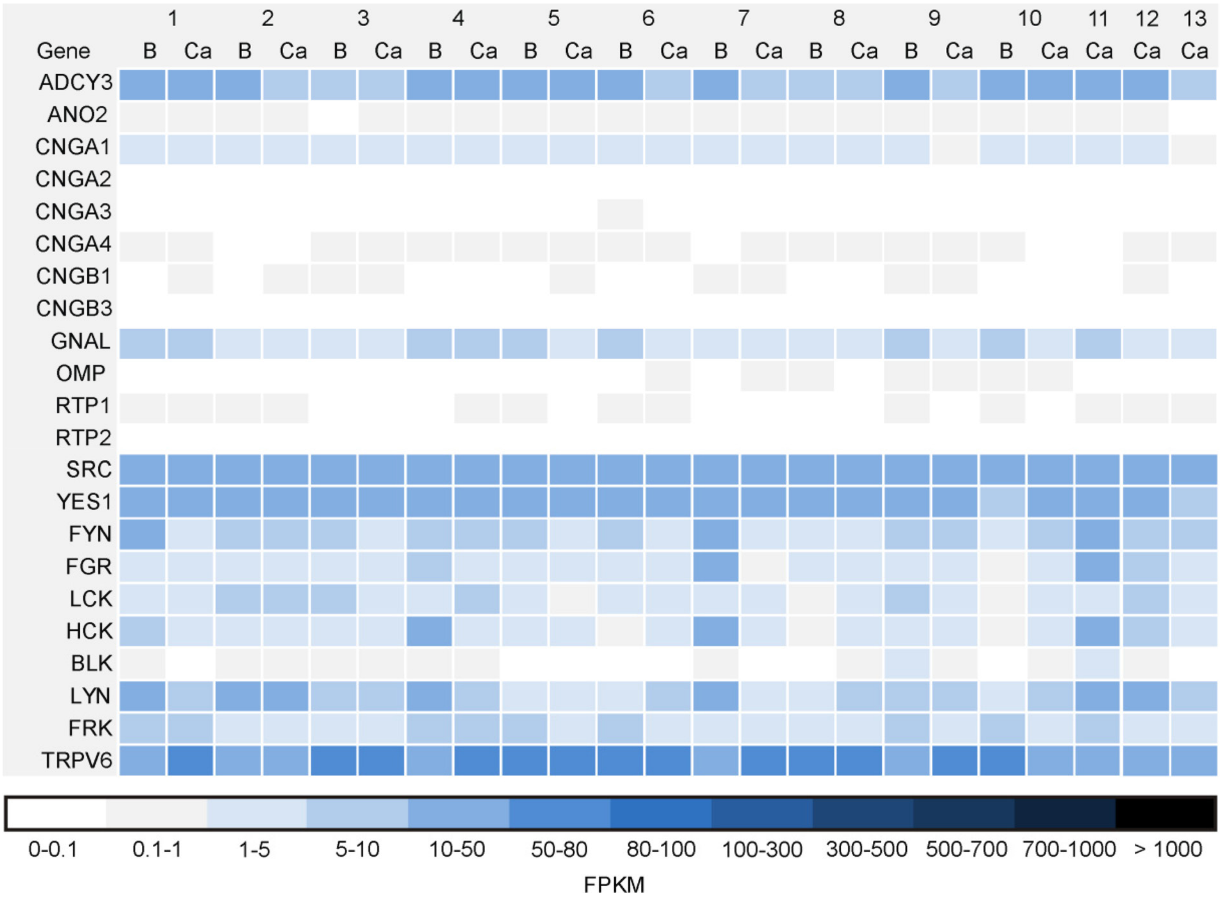

Supplementary Figure S2: Expression profile of olfactory signal transduction components in benign prostatic and PCa tissue as determined by RNA-Seq. Expression distribution of the olfactory signaling components adenylyl cyclase III (ADCY3), calcium-activated chloride channel (ANO2), cyclic nucleotide gated ion channel subunits (CNGA1, CNGA2, CNG3, CNGA4, CNGB1 and CNGB3), olfactory G-protein subunit $G\alpha_{olf}$ (GNAL), olfactory marker protein (OMP), the accessory proteins receptor-transporting proteins (RTP1 and RTP2), members of the src-kinase family (SRC, YES1, FYN, FGR, LCK, HCK, BLK, LYN and FRK) and the transient receptor potential channel V6 (TRPV6) in all analyzed benign prostatic (B) and PCa (Ca) tissues (P1-P13). In addition, three self-generated data sets of prostate PCa tissue were analyzed (P11-P13). Black and dark blue represent high transcript expression, light blue and white indicate low to no detectable transcript expression.

Specimen	OR51E1 p-value	q-value
P1	0.08	0.38
P2	0.07	0.39
P3	0.49	0.74
**P4**	**$3.45x10^{-7}$**	**$3.44x10^{-5}$**
P5	0.68	0.94
P6	0.72	0.95
P7	0.018	0.14
P8	0.54	0.80
P9	0.28	0.63
**P10**	**$1.78x10^{-15}$**	**$7.19x10^{-13}$**

Specimen	OR51E2 p-value	q-value
P1	0.38	0.79
P2	0.15	0.56
**P3**	**0.001**	**0.02**
**P4**	**$6.02x10^{-6}$**	**$4.23x10^{-4}$**
**P5**	**$3.81x10^{-8}$**	**$5.05x10^{-6}$**
**P6**	**$3.49x10^{-11}$**	**$3.15x10^{-8}$**
P7	0.65	0.92
P8	0.27	0.60
**P9**	**$1x10^{-4}$**	**$4.74x10^{-3}$**
**P10**	**$<1x10^{-15}$**	**$<1x10^{-15}$**

Supplementary Figure S3: Statistical analysis of differential OR expression in prostate tissue. Differential expression analysis of OR51E1 (left) and OR51E2 (right) between benign prostatic and PCa tissue (P1-P10) using Cuffdiff v.1.3.0. Shown are the uncorrected p-values and the false discovery rate (FDR)-adjusted p-values (q-values). Significant differences in expression are highlighted in red.

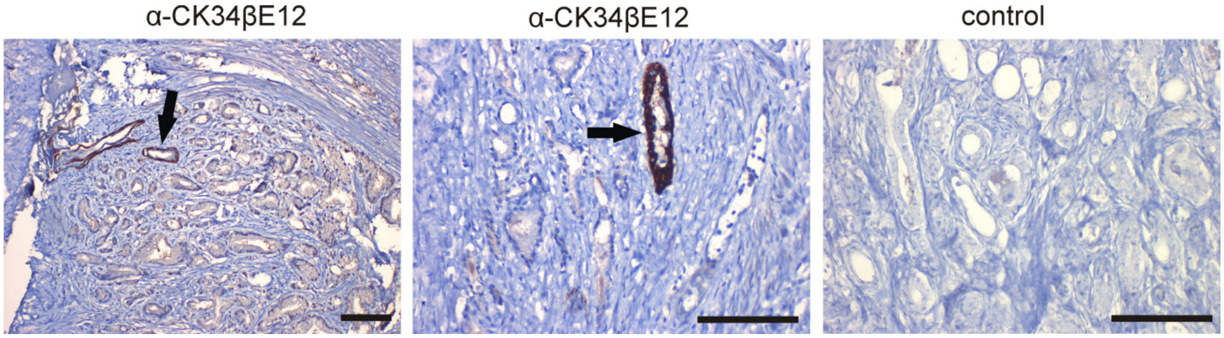

Supplementary Figure S4: Control IHC staining of human prostate tissue. (Left and middle) CK34βE12-positive glands in benign prostatic tissue. IHC of basal epithelial cells to identify benign prostatic prostate tissue using an α-CK34βE12 antibody. Black arrows indicate CK34βE12-positive prostate glands. (Right) The control showed no unspecific binding of the secondary antibody in moderately differentiated carcinoma (P14). Protein expression is visualized using DAB chromogenic staining. Tissue architecture is illustrated by co-staining with HE. Scale bar: 100 μm.

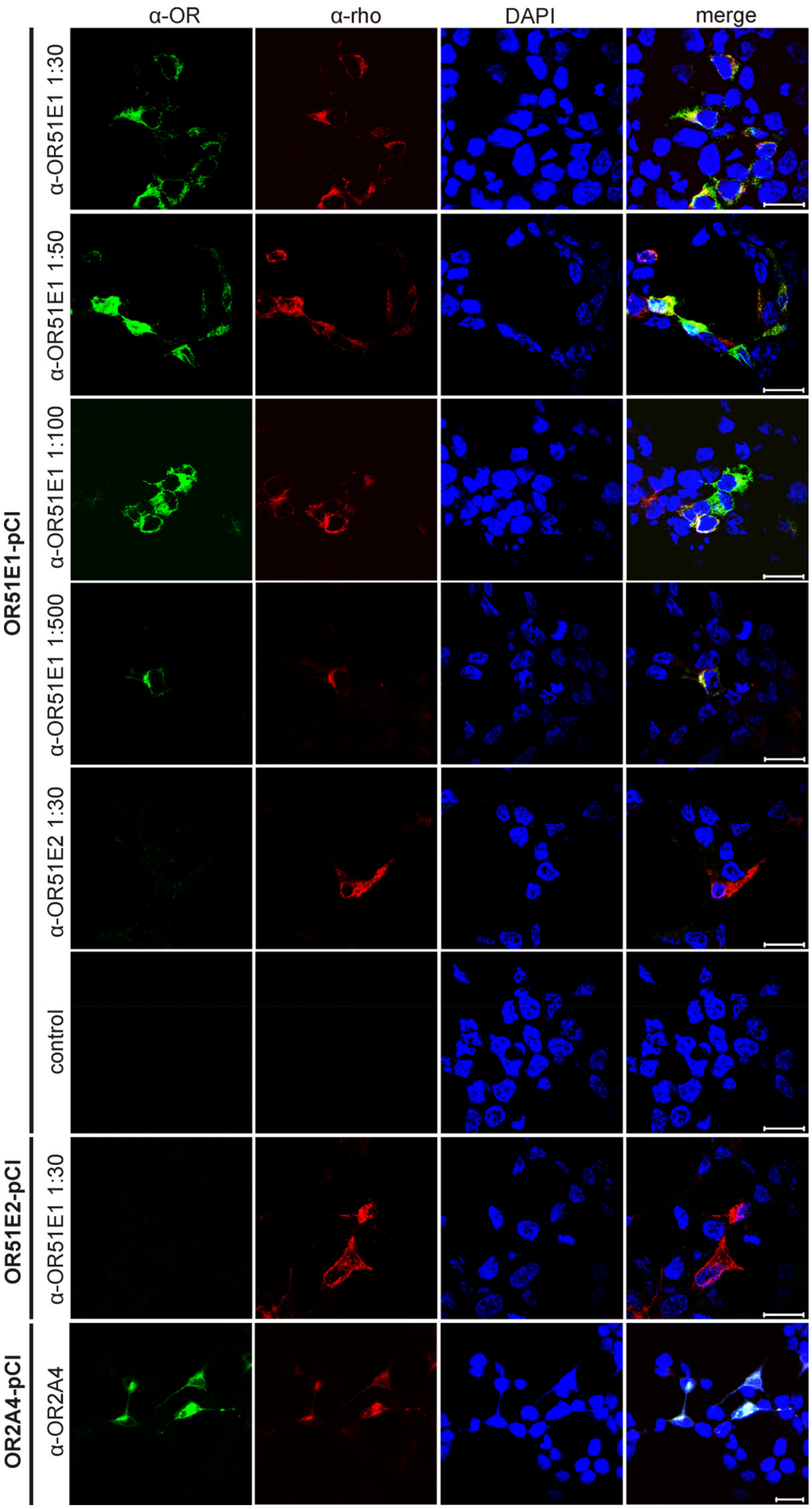

Supplementary Figure S5: IF control staining of OR-transfected Hana3A cells. IF staining of heterologously expressed OR51E1 (OR51E1-pCI), OR51E2 (OR51E2-pCI) and OR2A4 (OR2A4-pCI) in Hana3A cells using α-OR51E1 (dilution: 1:30, 1:50, 1:100 and 1:500), α-OR51E2 (dilution: 1:30) or α-OR2A4 (dilution: 1:100) specific antibodies (green) and α-rho antibody (red; dilution: 1:100). DAPI (blue) was used to visualize the nuclei of Hana3A cells. The control excluded nonspecific binding of the secondary antibodies Goat anti-Rabbit IgG Alexa Fluor® 488 and Goat anti-Mouse IgG Alexa Fluor® 546. Scale: 20 µm.

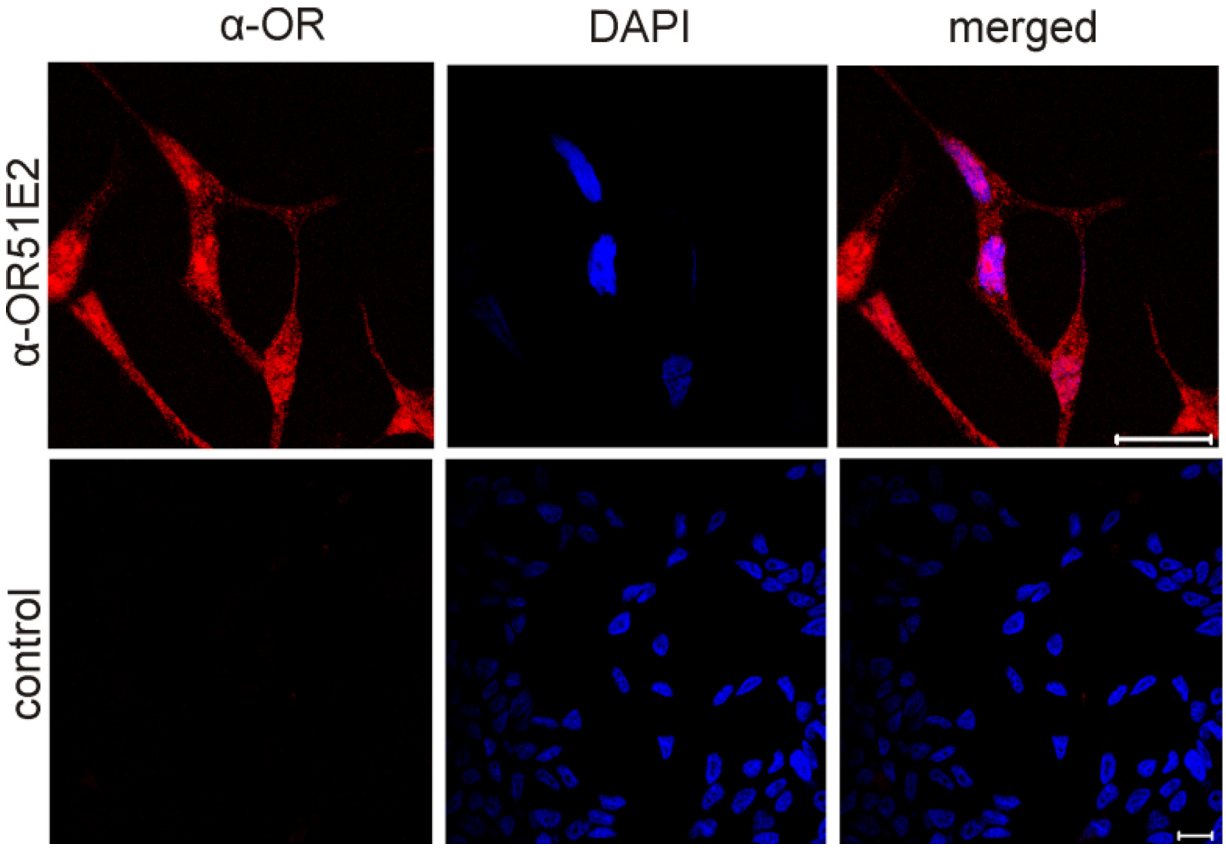

Supplementary Figure S6: IF control staining of LNCaP cells. (Top) IF to validate the protein expression of OR51E2 (red) in LNCaP cells. (Bottom) The control showed no unspecific binding of the secondary antibody. Nuclei were stained with DAPI (blue). Scale: 20 µm.

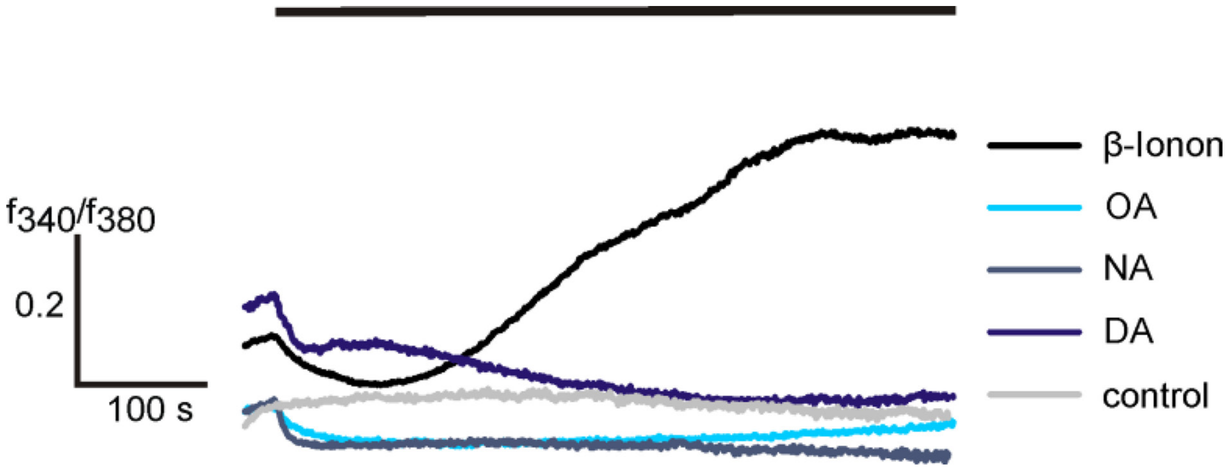

Supplementary Figure S7: The effect of different OR51E1-agonists on intracellular calcium levels in LNCaP cells using Calcium Imaging. Shown are representative calcium imaging traces (f_{340}/f_{380}) during the application of OA (1 mM), NA (1 mM) and DA (1 mM) for 10 minutes. The odorants and the control showed no effect on the intracellular calcium concentration of LNCaP cells. The known OR51E2 agonist β-ionone (500 µM) served as the positive control. Bar indicates the stimulus duration.

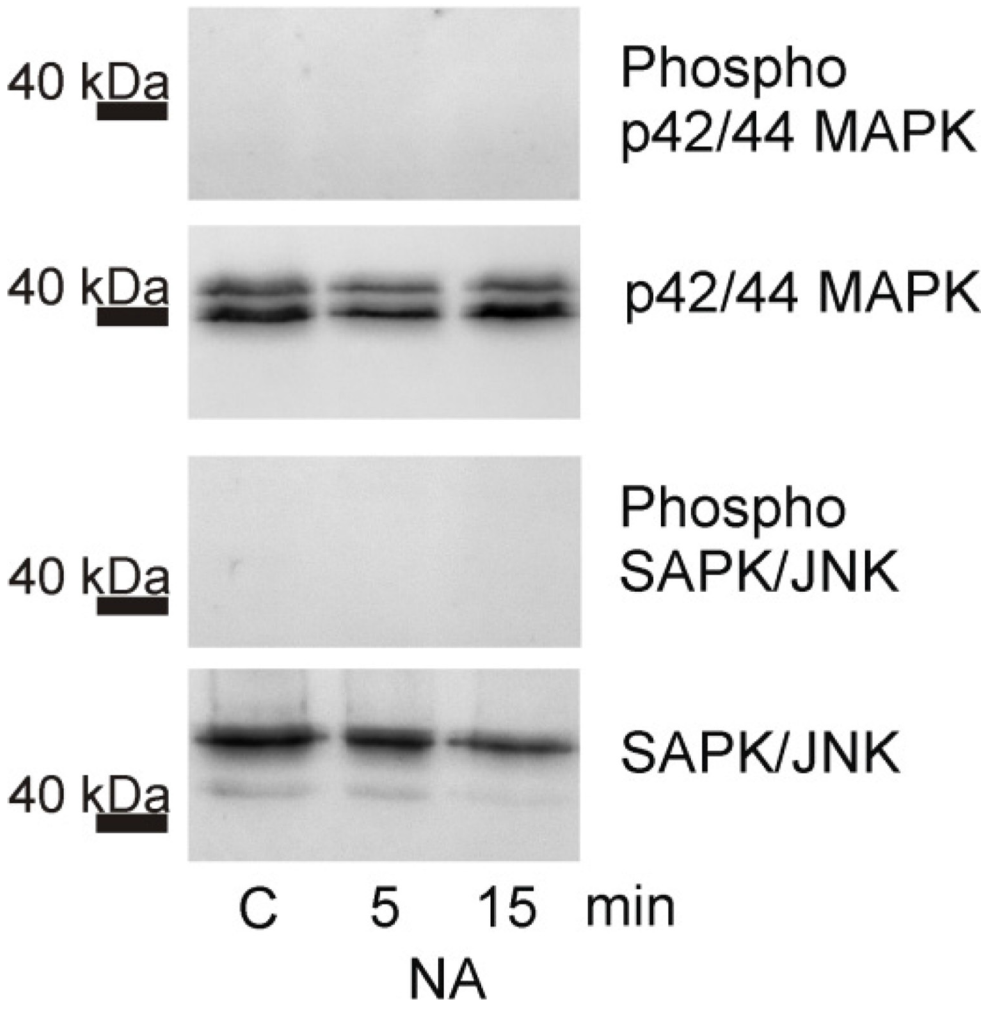

Supplementary Figure S8: Determination of the phosphorylation levels of p42/44 MAPK and SAPK/JNK upon NA in LNCaP cells. Western blot analysis to investigate the phosphorylation of p42/44 MAPK and JNK/SAPK in LNCaP cells after 5 min or 15 min stimulations with NA (300 µM) or control. Determination of the total amounts of p42/44 MAPK and JNK/SAPK served as controls.

Supplementary Table S1: Clinical data for prostate tissue specimens used in this study

See Supplementary File 1

Supplementary Table S2: Primer sequences used for RT-PCR

See Supplementary File 1
