# Supplementary material for: The activation of OR51E1 causes growth suppression of human prostate cancer cells
Source: Oncotarget. 2016 Jun 21;7(30):48231–49. doi: 10.18632/oncotarget.10197 (PMC5217014; doi:10.18632/oncotarget.10197)
Supplement: Supplementary file 2 [file oncotarget-07-48231-s002.pdf]

Tab. S1: Clinical data for prostate tissue specimens used in this study.

Specimen	Method	Histology	Gleason Score	Tumor stage	remarks
P11	RNA-Seq	poorly differentiated adenocarcinoma	4 + 3	pT3a	pN0 L0 V0 R1
P12	RNA-Seq	poorly differentiated adenocarcinoma	4 + 4	pT3a	pN0 Pn1 R0
P13	RNA-Seq	poorly differentiated adenocarcinoma	4 + 4	pT3a	pN0 L1 V0 Pn1 R0
PCa tissue	RT-PCR	poorly differentiated adenocarcinoma	3 + 4	pT2c	pN0 Pn1 L0 V0 R0
benign tissue	RT-PCR	/	/	/	benign prostatic hyperplasia questionable
P14	IHC	moderately differentiated adenocarcinoma	3 + 4	pT1a	
P15	IHC	well differentiated adenocarcinoma	3 + 3	pT1a	

Tab. S2: Primer sequences used for RT-PCR.

Gene	Primer sequence (5'-3')
OR51E1-forward	CCGCCATTGGCCTGGACTCA
OR51E1-reverse	CCAAGATGACGGGCAGCGGA
OR51E2-forward (LNCaP)	CTGAAAGGAACAGGCCGAAC
OR51E2-reverse	GACCACGATGCAGTTTCCAA
OR51E2-forward (prostate)	ACTGCCTTCCAAGTCAGAGC
OR51E2-reverse	CTTGCCTCCCACAGCCTG
OR2A4/7-forward (LNCaP)	GCCTACGCCTGCAACACGGT
OR2A4/7-reverse	TCGGAGGGGGTGGCAGATGG
OR2A4/7-forward (prostate)	GGTGCCCCGGATGCTGGTG
OR2A4/7-reverse	GGGGTGGCAGATGGCCACG
PSA-forward	AGTGCGAGAAGCATTCCCAA
PSA-reverse	TTTGGGGTCAAGACATCTGGC
AR-forward	GCCTGTTGAACTCTTCTGAGC
AR-reverse	GCTGTGAAGGTTGCTGTTCCTC
KRT8-forward	CTGGAGGCCGCCATTGCAGAT
KRT8-reverse	CAGACACCAGCTTCCCATCACG
KRT18-forward	TGAGACGACGCTCACAGAGCTGA
KRT18-reverse	TATCCGGCGGGTGGTGGTCTTTT
GAPDH-forward	ACCACAGTCCATGCCATCAC
GAPDH-reverse	TCCCACCACCCTGTTGCTGTA
TBP-forward	TATAATCCCAAGCGGTTTGC
TBP-reverse	GCTGGAAAACCCAACTTCTG
